# ITO-Based Electrically Tunable Metasurface for Active Control of Light Transmission

**DOI:** 10.3390/nano14191606

**Published:** 2024-10-05

**Authors:** Ruize Ma, Yu Mao, Peiyang Li, Dong Li, Dandan Wen

**Affiliations:** 1 Key Laboratory of Light Field Manipulation and Information Acquisition, Ministry of Industry and Information Technology, Xi’an 710129, China; mrz581@mail.nwpu.edu.cn (R.M.); myphy@mail.nwpu.edu.cn (Y.M.); 2022265001@mail.nwpu.edu.cn (P.L.); 2 Shaanxi Key Laboratory of Optical Information Technology, School of Physical Science and Technology, Northwestern Polytechnical University, Xi’an 710129, China

**Keywords:** Metasurface, Indium Tin Oxide, Multipole expansion, Active control spectrum

## Abstract

In recent years, the rapid development of dynamically tunable metasurfaces has provided a new avenue for flexible control of optical properties. This paper introduces a transmission-type electrically tunable metasurface, employing a series of subwavelength-scale silicon (Si) nanoring structures with an intermediate layer of Al_2_O_3_-ITO-Al_2_O_3_. This design allows the metasurface to induce strong Mie resonance when transverse electric (TE) waves are normally incident. When a bias voltage is applied, the interaction between light and matter is enhanced due to the formation of an electron accumulation layer at the ITO-Al_2_O_3_ interface, thereby altering the resonance characteristics of the metasurface. This design not only avoids the absorption loss of metal nanostructures and has a large modulation depth, but also shows compatibility with complementary metal oxide semiconductor (CMOS) technology.

## 1. Introduction

Metasurfaces are two-dimensional nanostructured surfaces that enable versatile wavefront control of light, and play a significant role in controlling the amplitude, phase, and polarization of reflected/transmitted light [1,2,3,4,5]. In addition to being significantly thinner than conventional optical elements, metasurfaces exhibit heightened sensitivity to variations in the dielectric environment, particularly to those due to resonances [6]. This sensitivity is further enhanced by employing field-concentrating designs. As a result, these metasurfaces allow for dynamic tuning, which can be achieved through various methods, such as electrical, optical, mechanical, and phase-change controls. One approach to facilitating this tunability is by incorporating plasmonic metasurfaces with active materials. By appropriately designing metasurfaces and selecting suitable tunable materials, dynamic control over physical parameters such as the phase and amplitude of light can be achieved [7]. Currently, commonly used tunable materials include liquid crystals [8,9,10], conductive oxide materials [11], two-dimensional materials [12], and phase-change materials [13]. Indium tin oxide (ITO) has garnered significant attention as an active electro-optical material, primarily due to its substantial refractive index variation and broad tuning capability; it has been widely used in the active control of dielectric singularities [14], in broadband infrared metamaterial absorbers [15], and so on. This material also features an epsilon-near-zero (ENZ) frequency range, where its permittivity transitions from positive to negative. Additionally, ITO exhibits a rapid response time at near-infrared (NIR) wavelengths, notably including the 1.55 μm wavelength used in telecommunications. Huang et al. [16] experimentally demonstrated a gate-tunable metasurface capable of dynamically controlling the phase and amplitude of the reflected plane wave through electrical modulation. This tunability is achieved via field-effect modulation of the complex refractive index of conducting oxide layers, which are integrated into metasurface antenna elements arranged in a reflect-array configuration. By applying a 2.5 V gate bias, a phase shift of π and an approximately 30% variation in reflectance are realized. Park et al. [17] introduced an all-solid-state, electrically tunable metasurface array capable of generating a specific phase or performing a continuous phase sweep from 0 to 360° at a frequency of approximately 5.4 MHz, while simultaneously modulating the amplitude. A key innovation in their design is the incorporation of two independent control parameters—top and bottom gate voltages—within each nanoresonator. This configuration allows for precise and independent adjustment of the real and imaginary components of the reflection coefficient, enhancing the metasurface’s tunability and functionality. These metasurfaces successfully achieve dynamic regulation of the beam; however, most metasurfaces based on dynamically tunable structures are primarily of the metal–insulator–metal (MIM) type, with less amplitude modulation or lower modulation depth in the transmission spectrum.

In this paper, we present an ITO-integrated all-dielectric metasurface for amplitude modulation in the transmission mode. A series of subwavelength-scale nanorings with carefully chosen inner and outer radii and periodicity are employed. This structural design enables the metasurface to induce strong Mie resonances and leads to field confinement in the ITO layer with TE-polarized light. To evaluate the optical and electrical performance of the metasurface, we calculated the voltage-dependent carrier distribution profiles for ITO and silicon (Si) using the Poisson equation and the electron–hole drift-diffusion equation. These profiles are then used as input for optical simulations to accurately determine the transmission responses under an applied bias voltage. Further investigation reveals significant variations in the modulation depth of the transmission spectrum at different voltages by altering the inner diameter of the nanorings. This design not only achieves significant breakthroughs in performance but also provides new insights and methods for studying electrically tunable metasurfaces.

## 2. Materials and Methods

**Figure 1:**
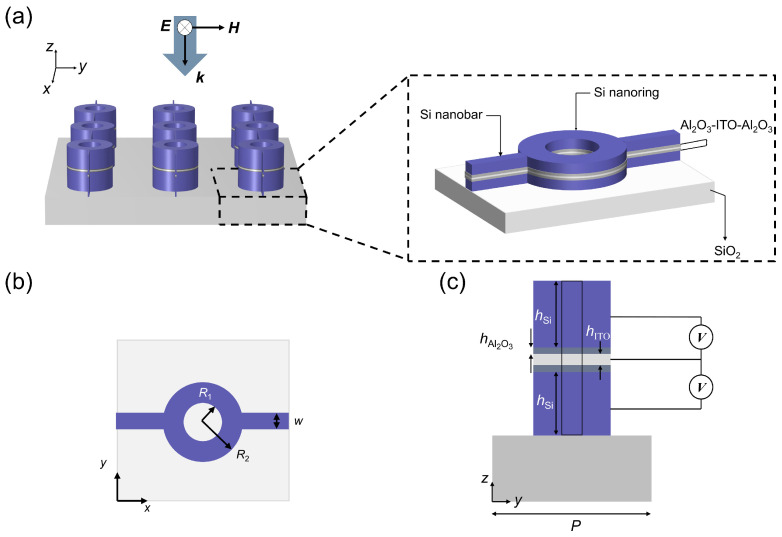
Schematic of the electrically tunable metasurface composed of Si nanorings. (**a**) Three-dimensional structure of the metasurface. The substrate is SiO_2_ and the Si nanorings have intermediate layers of Al_2_O_3_/ITO/Al_2_O_3_. Adjacent Si nanorings in the same column are connected by Si nanobars. By controlling the magnitude of the applied bias voltage, the metasurface can modulate the transmission spectrum over a significant range in the near-infrared wavelength band. (**b**) Top view (*x*-*y* plane) of the structural unit. The inner and outer radii of the rings are represented by *R*_1_ and *R*_2_, respectively, and the width of the nanobars is denoted as *w*. (**c**) Cross-sectional view (*y*-*z* plane) of the structural unit. The period of the structural unit is *P*, and the thickness of both the upper and lower Si layers is *h*_Si_, while that of the upper and lower Al_2_O_3_ layers is *h*_Al2O3_. The thickness of the ITO layer is *h*_ITO_. Throughout this paper, the following parameters are kept constant: *w* = 20 nm, *P* = 600 nm, *h*_Si_ = 140 nm, *h*_Al2O3_ = 5 nm, *h*_ITO_ = 10 nm.

The electrically tunable metasurface is schematically shown in Figure 1. According to the charge conservation, the Si-Al_2_O_3_-ITO heterojunction can be regarded as a non-ideal parallel plate capacitor composed of the ITO layer and the Si layer acting as semiconductor plates, with equal and opposite induced charges [18]. Herein, the ITO layer is degenerately n-type doped with a background carrier concentration of 3 × 10^20^ cm^−3^, while the Si layer is heavily n-type doped with a background carrier concentration [19] of 1 × 10^19^ cm^−3^. The Si layer serves as the electrode which ensures the presence of sufficient free electrons to conduct current through the introduction of impurities (e.g., arsenic or phosphorus), thus enabling it to be utilized as a bias electrode for applying voltage to the ITO layer. To avoid applying bias individually to each nanoring, Si nanorings in the same column are connected by nanobars in the *x*-direction and controlled by the same voltage from one side. The strong Mie resonance is excited with TE- or TM-polarized light (polarized along or perpendicular to the nanobars) and is almost unaffected by the nanobars. Upon applying voltage to the metasurface, the formation of an electron accumulation layer at the ITO-Al_2_O_3_ interface enhances the interaction between light and matter, thereby altering the resonant properties of the metasurface. In this design, the refractive index of ITO is simulated using the Drude model [20], while the refractive indices of Si, Al_2_O_3_, and SiO_2_ are obtained from experimental data [20].

**Figure 2:**
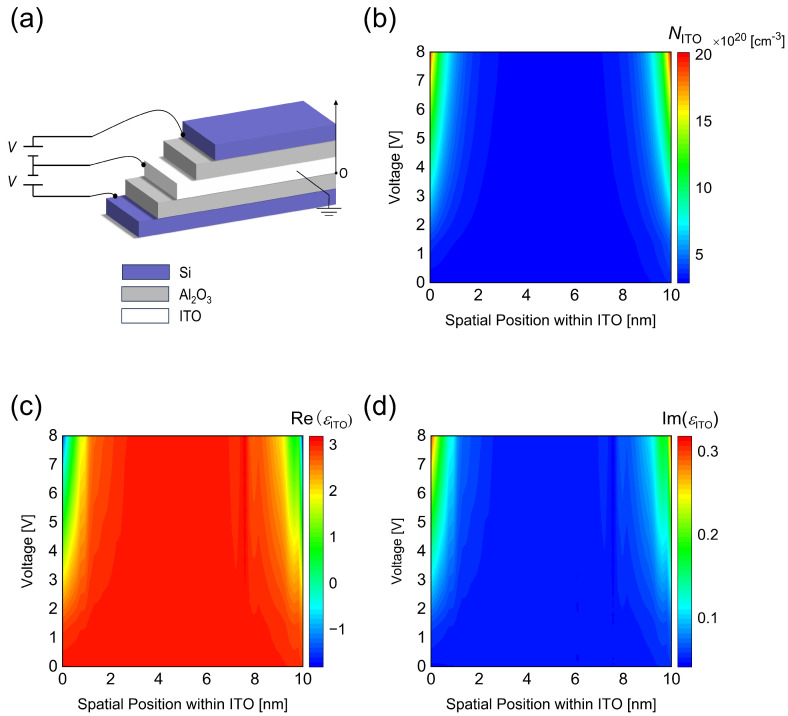
Schematic representation of biasing for the electrically tunable metasurface, and simulation of carrier concentration and dielectric constant of ITO. (**a**) Schematic diagram of the biasing scheme, where the voltage is applied to the Si layer and the ITO layer is grounded. (**b**) Variation of electron concentration in the ITO accumulation layer as a function of applied bias voltage and distance from the ITO-Al_2_O_3_ interface. (**c**) Real part and (**d**) imaginary part of the dielectric constant of ITO as functions of voltage and distance from the ITO-Al_2_O_3_ interface at the wavelength of 1038 nm, with an applied bias voltage ranging from 0 V to 8 V.

To determine the effect of applied voltages on Mie resonance modes, we first simulate the carrier concentration distribution of ITO under different bias voltages. As shown in Figure 2a, the same positive voltage is applied to the top and bottom Si layers, while the ITO layer is grounded (diagram of applying a voltage from one side of the metasurface is shown in Supplementary Note S1). For different bias voltages, the spatial distribution of carrier concentration in the accumulation layer can be calculated by solving the Poisson equation and the electron–hole drift-diffusion equation. As depicted in Figure 2b, increasing the applied bias voltage from 0 V to 8 V results in an increase in carrier concentration at the ITO-Al_2_O_3_ interface from 3 × 10^20^ cm^−3^ to 2.1 × 10^21^ cm^−3^. The accumulation of carrier concentration at the ITO-Al_2_O_3_ interface decays exponentially with distance, leading to an effective thickness of the accumulation layer of approximately ≈ 1 nm. The slight accumulation of carrier concentration at the ITO-Al_2_O_3_ interface under zero bias voltage is attributed to the difference in the work function of doped Si and ITO, resulting in a band-bending effect. In this model, the dielectric constant [21] of ITO is 9.3, the bandgap [22] is 2.8 eV, and the work function [16] is 6.4 eV.

After solving the Poisson equation and the drift-diffusion equation, the spatial distribution of carrier concentration within the active region of ITO is transformed into a spatial distribution of dielectric constant using a dispersion model, which can be subsequently incorporated into optical simulations. The dielectric constant of ITO can be obtained from the Drude model [20]:

(1) $$\varepsilon_{\mathrm{ITO}}=\varepsilon_{\infty }-\omega_{p}^{2}/(\omega^{2}+i\omega \Gamma )$$

(2) $$\omega_{p}^{2}=\frac{Ne^{2}}{\varepsilon_{0}m^{*}}$$

where *ε*_∞_ is the high-frequency dielectric constant, *Γ* is the damping constant, and m* is the effective mass of electrons, with *m*_0_ representing the electron rest mass [23]. Here we have values of *ε*_∞_ = 3.9, *Γ* = 100 THz, and m* = 0.35 × *m*_0_, where *m*_0_ represents the electron’s rest mass.

## 3. Results

**Figure 3:**
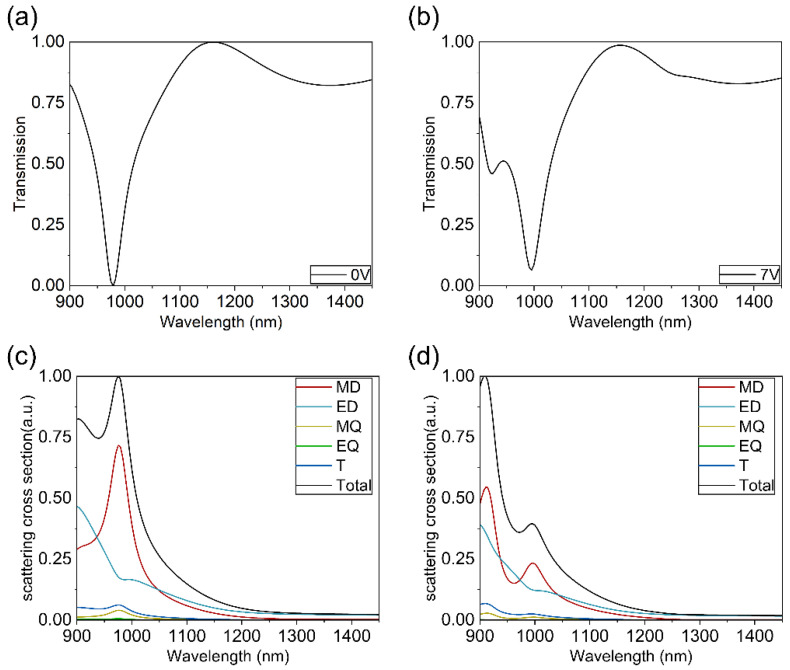
Transmission spectrum of the metasurface and corresponding scattering cross-sections obtained at different voltages. In this part, we set the inner radius and outer radius of nanoring units to be 80 nm and 160 nm, respectively. (**a**) Transmission spectrum of the metasurface under TE-polarized incident light without an applied bias voltage and (**b**) with a 7 V applied bias voltage. (**c**) Calculated scattered cross-sections for electromagnetic multipoles of the metamaterial array without applied bias voltage and (**d**) with a 7 V applied bias voltage. The plot displays the first five multipoles: magnetic dipole (MD), electric dipole (ED), toroidal dipole (T), electric quadrupole (EQ), and magnetic quadrupole (MQ).

As shown in Figure 3, to demonstrate the effect of bias voltage on the metasurface, we employ finite-difference time-domain (FDTD) simulations to obtain the transmission spectrum under 0 V and 7 V bias voltage. For ease of calculation, we divide the ITO layer into finite intervals, set the average electron concentration in an interval as the electron concentration of the interval, and calculate the dispersion curve by the Drude model. We use nanoring units with an inner radius *R*_1_ = 80 nm and an outer radius *R*_2_ = 160 nm, with TE-polarized incident light with wavelengths of 900–1450 nm. The results show that without applied bias voltage, the transmission spectrum of the metasurface exhibits a transmission dip at a wavelength of 978 nm. When a bias voltage of 7 V is applied, the carrier concentration in ITO increases, forming an ENZ (epsilon-near-zero) region at the Al_2_O_3_-ITO interface. The electric field is significantly enhanced within the accumulation layer, resulting in a noticeable change in the resonance characteristics of the metasurface, leading to the transmission spectrum splitting into two valleys with lowest transmittance values of 995.3 nm and 924.8 nm.

For nanoparticles composed of high-index materials such as Si, the resonances are predominantly induced by Mie scattering. To analyze the changes in modes before and after applying voltage, we calculated the far-field scattering of this metasurface structure using multipole expansion. The center of the decomposition is located at *x*, *y* = 0 μm, and *z* = 0.15 μm. The decomposition takes into account the spherical ring and the bars. Far-field scattering refers to the electromagnetic radiation re-emitted by various multipole modes within the nanostructure under plane wave illumination in periodic arrangements [24]. The predominant multipole modes include electric dipole (ED), magnetic dipole (MD), electric quadrupole (EQ), magnetic quadrupole (MQ), and toroidal dipole (T) modes [25] (see Supplementary Note S2). In a dielectric nanoparticle, magnetic dipole resonance (MD) arises from the excitation of a specific electromagnetic mode within the particle, characterized by a circular displacement current of the electric field. As shown in Figure 3c, without applied voltage, the MD mode dominates the transmission peak at 978 nm. Meanwhile, as depicted in Figure 3d, after applying bias voltage, the resonance peaks observed at wavelengths of 924 nm and 995 nm are primarily influenced by the MD mode (more analysis of spectral splitting is provided in Supplementary Note S3).

**Figure 4:**
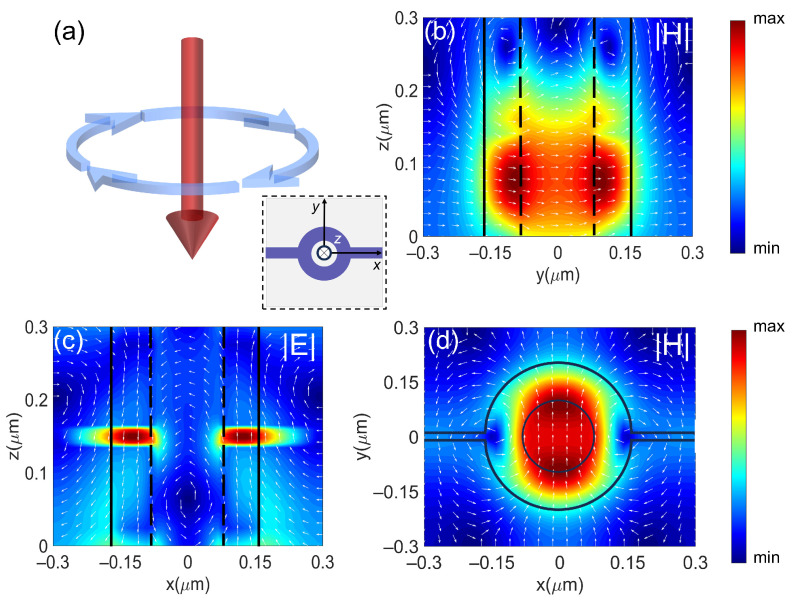
(**a**) Charge distribution and current flow of the MD mode. (**b**) Near-field distribution of the magnetic field component (|**H**|) (cross-section in the *y*-*z* plane) at a wavelength of 924 nm under 7 V bias voltage, where the outline of the nanoring is represented by dark dashed lines and the field directions are represented by white arrows. (**c**) Near-field distribution of the electric field component (|**E**|) (cross-section in the *x*-*z* plane) and displacement current at a wavelength of 924 nm under 7 V bias voltage. (**d**) Magnetic field distribution in the *x*-*y* plane at a wavelength of 924 nm under 7 V bias voltage. Max represents the maximum of *E*/*E*_0_ and *H*/*H*_0_, and min represents the minimum of *E*/*E*_0_ and *H*/*H*_0_.

To verify the excitation of the MD mode, we simulate the field distributions inside the metasurface unit. Figure 4a is a schematic of the magnetic dipole pattern, which can be represented by a ring current (blue arrow). Currents flow in a ring path, creating a magnetic field perpendicular to the plane of the ring current (red arrow). Figure 4b,c illustrate the normalized magnetic field intensity (cross-section in the *y*-*z* plane) and electric field intensity (cross-section in the *x*-*z* plane), respectively, for *λ* = 924 nm and a bias voltage of 7 V under TE-polarized incident light. Figure 4d presents the magnetic field distribution in the *x*-*y* plane. The white arrows in Figure 4d represent displacement currents, while the dark dashed lines indicate the structural outline. From the magnetic field distribution in Figure 4d, we observe a strong magnetic field distribution within the nanoring, accompanied by circulating displacement currents inside the nanoring, as shown in Figure 4c. These results verify that the Mie resonance at *λ* = 924 nm is induced by the magnetic dipole mode. The near-field distribution at 924 nm without the bias and the in the case of 995 nm (with/without the bias) are shown in Supplementary Note S4.

**Figure 5:**
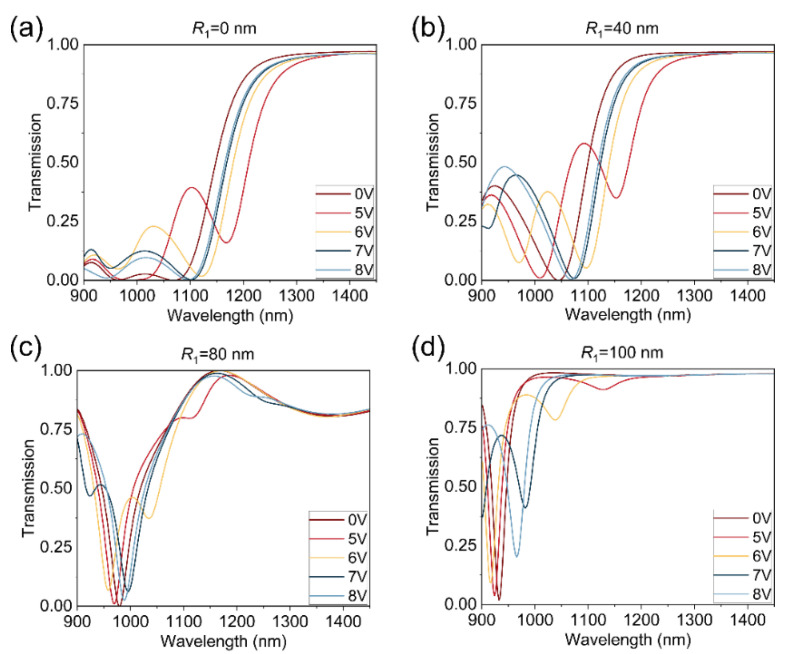
Transmission spectrum of the electrically tunable metasurface under different voltages, with inner radius *R*_1_ = 0 nm, 40 nm, 80 nm, and 100 nm, and outer radius *R*_2_ = 160 nm. The light source is a normally incident TE-polarized plane wave.

To investigate the relationship between the transmission spectrum and structural parameters, we conduct a parameter sweep over the inner radius of the nanorings. While keeping the outer radius constant at 160 nm, we vary the inner radius to 0 nm, 40 nm, 80 nm, and 100 nm, with bias voltages of 0 V, 5 V, 6 V, 7 V, and 8 V, respectively. The incident light is TE-polarized. The transmission spectrum of the metasurface as a function of voltage and inner radius are shown in Figure 5. By increasing the inner radius, we observe that, similar to Figure 3b, the transmission spectra still exhibit two dips under different voltages, one of which is more prominent while the other peak is smaller. It is noteworthy that the quality factor (Q-value) of the larger transmission dip increases with increasing inner radius, and the resonance region gradually blueshifts. Compared with the periodic Si disk structure, the electric field of the periodic Si ring structure is more localized, resulting in a higher Q value of the transmission spectrum and a higher modulation depth, as shown in Figure 5 (more details about modulation depth calculations are provided in Supplementary Note S5). When the inner radius is 40 nm, the metasurface exhibits a large range of resonance peak shifts with increasing voltage. As the inner radius continues to increase, the resonance peak shift of the transmission spectrum decreases with increasing applied voltage, while the quality factor gradually increases. When the inner radius reaches 100 nm, the disparity between the inner and outer radius is small, and the electric field becomes more localized in the accumulation layer of ITO in the nanorings, resulting in the narrowest spectral bandwidth. Since the distribution of Mie resonance modes is directly related to the radius difference of the nanoring array, careful selection of geometric factors of the nanoring array and applied voltage magnitude allows for the obtaining of the desired wavelength and the desired depth of the transmission dip.

## 4. Conclusions

This paper introduces an innovative design of the electrically tunable metasurface consisting of a series of Si nanorings with ITO sandwiched between them on a glass substrate. By applying a certain voltage to the metasurface, the excitation condition of the magnetic dipole (MD) mode changes, thereby affecting the optical performance of the metasurface. Further studies have shown that significant electrical modulation of the transmission spectrum can be achieved by selecting the inner and outer radius as well as the period of the nanorings. This design not only avoids the absorption losses of metallic nanostructures, but also allows for dynamic control of the transmission spectrum rather than the reflection spectrum. The device also shows compatibility with CMOS technology. We envisage that the device can be fabricated as silicon-based sensors and spectral reconstruction filters.

## Data Availability

The data that support the findings of this study are available from the corresponding authors upon reasonable request.

## Supplementary Materials

The following supporting information can be downloaded at https://www.mdpi.com/article/10.3390/nano14191606/s1, Figure S1. Diagram of applying a voltage from one side of the metasurface. Figure S2. Transmission spectrum of the metasurface with one accumulation layer under TE-polarized incident light with a 7 V applied bias voltage. Figure S3. (a) The structure diagram in *x*-*y* plane. (b) Near-field distribution of the magnetic field component (|H|) (cross-section in the *y*-*z* plane) at a wavelength of 995 nm under 7 V bias voltage, where the outline of the nanoring is represented by dark dashed lines, the field directions is represented by white arrows. (c) Near-field distribution of the electric field component (|E|) (cross-section in the *x*-*z* plane) and displacement current at a wavelength of 995 nm under 7 V bias voltage. (d) Magnetic field distribution in the *x*-*y* plane at a wavelength of 995 nm under 7 V bias voltage. Figure S4. (a) and (d) Near-field distribution of the electric field component (|E|) (cross-section in the *x*-*z* plane) and displacement current at a wavelength of 924 nm and 995 nm under 7 V bias voltage. (b) and (e) Near-field distribution of the magnetic field component (|H|) (cross-section in the *y*-*z* plane) at a wavelength of 924 nm and 995 nm under 7 V bias voltage, where the outline of the nanoring is represented by dark dashed lines, the field directions is represented by white arrows. (c) and (f) Magnetic field distribution in the *x*-*y* plane at a wavelength of 924 nm and 995 nm under 7 V bias voltage. Figure S5. The modulation depth under different voltages when R1 = 0.04 μm. Figure S6. Variation of (a) hole concentration (b) electron concentration in the Si accumulation layer as a function of applied a bias voltage and distance from the Si-Al_2_O_3_ interface. Figure S7. The real and imaginary parts of the Si permittivity located near the Si-Al_2_O_3_ surface at 8 V voltage. Figure S8. Dispersion curve of ITO at the closest layer to the Al_2_O_3_ layer (a) with a 0 V voltage and (b) with a 7 V applied bias voltage. Table S1. Comparison of proposed and reported metasurfaces for dynamic regulation. Refs [19,20,24,26,27,28,29,30,31] are cited in the supplementary materials.
