# Supplementary material for: ITO-Based Electrically Tunable Metasurface for Active Control of Light Transmission
Source: Nanomaterials (Basel). 2024 Oct 5;14(19):1606. doi: 10.3390/nano14191606 (PMC11479147; doi:10.3390/nano14191606)
Supplement: Supplementary file 1 [file nanomaterials-14-01606-s001.zip › nanomaterials-3217347-supplementary.pdf]

Supplementary Information for

ITO-Based Electrically Tunable Metasurface for Active Control of Light Transmission

Authors:

Ruize Ma [1,2], Yu Mao [1,2], Peiyang Li [1,2], Dong Li [1,2,*] and Dandan Wen [1,2,*]

Affiliations:

1 Key Laboratory of Light Field Manipulation and Information Acquisition, Ministry of Industry and Information Technology, Xi'an 710129, China; mrz581@mail.nwpu.edu.cn (R.M.); myphy@mail.nwpu.edu.cn (Y.M.); 2022265001@mail.nwpu.edu.cn (P.L.)

2 Shaanxi Key Laboratory of Optical Information Technology, School of Physical Science and Technology, Northwestern Polytechnical University, Xi'an 710129, China

* Correspondence: dongli@nwpu.edu.cn (D.L.); dandanwen@nwpu.edu.cn (D.W.)

Supplementary Note S1. Diagram of applying a voltage from one side of the metasurface.

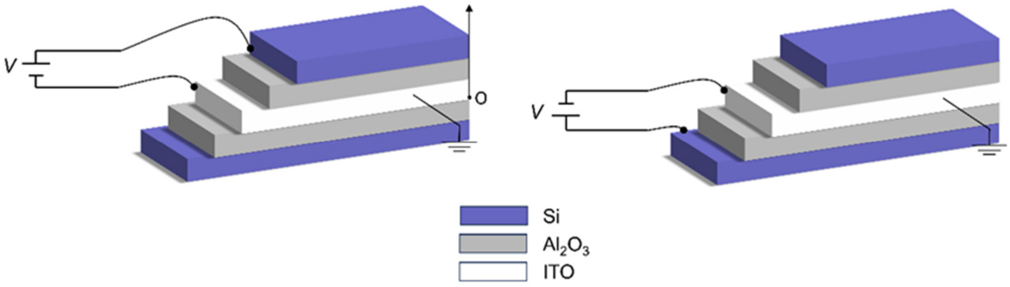

Figure S1. Diagram of applying a voltage from one side of the metasurface.

Supplementary Note S2. Multipolar expansion.

First, we describe the expression of multipole expansion reported in [1]. Assume that a resonator in free space is illuminated by a plane wave with an electric field amplitude of $|\boldsymbol{E}_{inc}| = \boldsymbol{E}_0$ at the

frequency f. The basis is a Cartesian coordinate system, and a position vector can be defined as $\boldsymbol{r}$ = ($\boldsymbol{x}$, $\boldsymbol{y}$, $\boldsymbol{z}$). When incident light illuminates the resonator, the induced current density distributions $\boldsymbol{J}$ can be obtained from the electric field distributions $\boldsymbol{E}$ by:

$$\boldsymbol{J} = -i\omega\epsilon_0(n^2 - 1)\boldsymbol{E} \tag{S1}$$

, where ω is the angular frequency, ϵ_0 is the permittivity of free space, and n is the refractive index of the resonator. Note that $\boldsymbol{J}$ corresponds to displacement current distributions in the case of dielectric Mie resonators. The multipole moments, that is, electric dipole $\boldsymbol{P}$, magnetic dipole $\boldsymbol{M}$, toroidal dipole $\boldsymbol{T}$, electric quadrupole $\boldsymbol{EQ}$, magnetic quadrupole $\boldsymbol{MQ}$:

Electric dipole moment: $\boldsymbol{P} = \frac{1}{i\omega}\int \boldsymbol{J}\, d^3r$

Magnetic dipole moment: $\boldsymbol{M} = \frac{1}{2c}\int (\boldsymbol{r}\times\boldsymbol{J})d^3r$

Toroidal dipole moment: $\boldsymbol{T} = \frac{1}{10c}\int [\boldsymbol{r}\cdot\boldsymbol{J}]\boldsymbol{r} - 2r^2\boldsymbol{J}]d^3r$

Electric quadrupole moment: $\boldsymbol{EQ} = \frac{1}{2i\omega}\int [r_\alpha J_\beta + r_\beta J_\alpha - \frac{2}{3}(\boldsymbol{r}\cdot\boldsymbol{J})\delta_{\alpha\beta}]d^3r$

Magnetic quadrupole moment: $\boldsymbol{MQ} = \frac{1}{3c}\int [(\boldsymbol{r}\times\boldsymbol{J})_\alpha r_\beta + (\boldsymbol{r}\times\boldsymbol{J})_\beta r_\alpha]d^3r$

The corresponding total scattering cross section is:

$$C_{sca}^{total} = \frac{k^4}{6\pi\epsilon_0^2 |E_0|^2}\left[\sum\left(|\boldsymbol{P}+ik\boldsymbol{T}|^2 + \left|\frac{\boldsymbol{M}}{c}\right|^2\right) + \frac{1}{120}\sum\left(|\boldsymbol{EQ}|^2 + \left|\frac{k\boldsymbol{MQ}}{c}\right|^2\right)\right] \tag{S2}$$

As can be seen, the total scattering cross section is a sum of partial scattering cross sections from different multipoles.

Supplementary Note S3. The explanation of the peak splitting using Coupled-Mode-Theory.

When a 7 V voltage is applied, the electron concentration of ITO deposits increases. The permittivity of ITO can be calculated by Drude model. In this case, the real part of the permittivity of ITO deposits is close to 0 and the electric field within the ITO accumulation layer is enhanced. Since the MD mode loop current passes through the ITO accumulation layer, the MD mode excited by Si is affected.

We have developed an analytical model to further understand the origin of the metasurface spectral splitting of double-layer ITO. The upper and lower accumulation layers affect the MD mode excited by Si rings respectively and generate two modified modes which we name as modified mode 1 and modified mode 2. Next, we use the Coupled-Mode-Theory (CMT)[2] to describe the spectral splitting induced by the coupling of the bilayers. In the absence of an external excitation source, the two modified modes $A = (A_1, A_2)^T$ evolve over time as $i\partial A/\partial t = HA$, where the Hamiltonian is given by:

$$H = \begin{pmatrix} \omega_0 & k \\ k & \omega_0 \end{pmatrix} - i\gamma \begin{pmatrix} 1 & e^{i\psi} \\ e^{-i\psi} & 1 \end{pmatrix} \tag{S3}$$

Here, k represents the near-field coupling between the two oscillators, γ is the radiation rate of the oscillators. Since the upper and lower accumulation layers are applied with the same voltage and the structure is mirror symmetric for $z = 0.15$ μm, we believe that the radiation effects of the two modified modes on the MD mode are exactly the same. At a specific wavelength, when the distance of the accumulation layer meets $\psi = kd$, the radiation effect of modified mode 1 on MD mode and that of modified mode 2 on MD mode cancel out exactly in the far field, resulting in spectral splitting. To test this theory, we apply a bias of 7 V in the case of a single bias. When TE

light is vertically incident on the metasurface, the resulting transmission spectrum show that the MD mode does not produce splitting under these conditions, thus proving that the spectral splitting is due to the dual correction modes cancelling each other out in the far field.

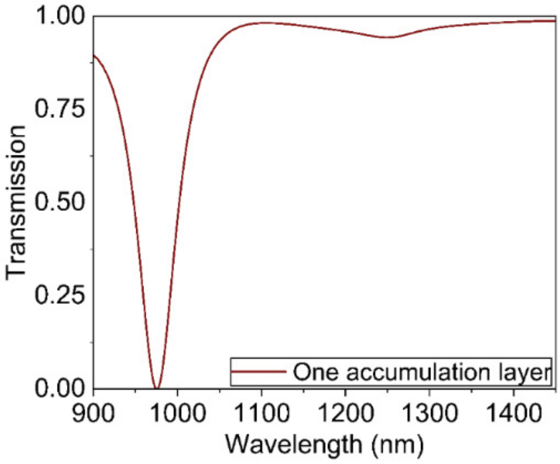

Figure S2. Transmission spectrum of the metasurface with one accumulation layer under TE-polarized incident light with a 7 V applied bias voltage.

Supplementary Note S4. The near-field distribution at 924 nm and 995 nm with/without the bias.

In Fig. 4 of the original manuscript, we plot the electromagnetic field distribution at the incident light wavelength of 924 nm. Here we further plot the electromagnetic field distribution at the incident wavelength of 995 nm. As shown in Fig. S3, the electromagnetic field distribution is similar to that at 924 nm, and there are also cyclic displacement currents inside the nanoring, thus confirming that the resonance at λ = 995 nm is also caused by the magnetic dipole mode.

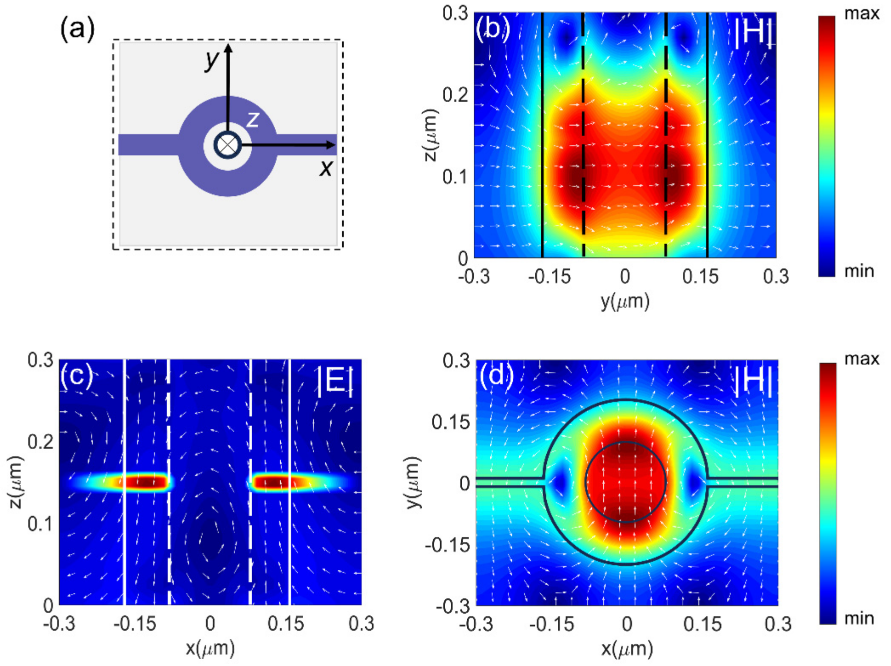

Figure S3. (a) The structure diagram in *x*-*y* plane. (b) Near-field distribution of the magnetic field component (|**H**|) (cross-section in the *y*-*z* plane) at a wavelength of 995 nm under 7 V bias voltage, where the outline of the nanoring is represented by dark dashed lines, the field directions is represented by white arrows. (c) Near-field distribution of the electric field component (|**E**|) (cross-section in the *x*-*z* plane) and displacement current at a wavelength of 995 nm under 7 V bias voltage. (d) Magnetic field distribution in the *x*-*y* plane at a wavelength of 995 nm under 7 V bias voltage.

Keeping the other structural parameters unchanged, we draw the electric field and magnetic field diagram of each cross section without applying voltage. Figures S4 (a) and (d) demonstrate that the Mie resonance remains a magnetic dipole mode when the incident light wavelengths are 924 nm and 995 nm. However, due to the resonance characteristics at different wavelengths and the variation in material dispersion parameters, the distribution of electric and magnetic fields in the magnetic dipole (MD) mode differs from that under the applied voltage.

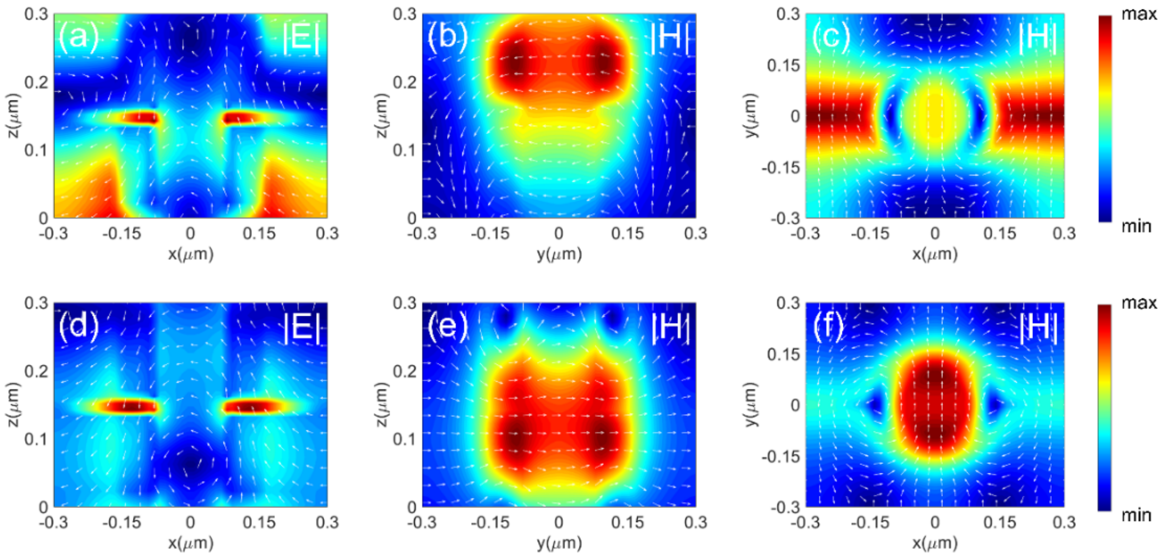

Figure S4. (a) and (d) Near-field distribution of the electric field component (|**E**|) (cross-section in the *x*-*z* plane) and displacement current at a wavelength of 924 nm and 995 nm under 7 V bias voltage. (b) and (e) Near-field distribution of the magnetic field component (|**H**|) (cross-section in the *y*-*z* plane) at a wavelength of 924 nm and 995 nm under 7 V bias voltage, where the outline of the nanoring is represented by dark dashed lines, the field directions is represented by white arrows. (c) and (f) Magnetic field distribution in the *x*-*y* plane at a wavelength of 924 nm and 995 nm under 7 V bias voltage.

Supplementary Note S5. The modulation depth under different voltages.

In the field of optics, modulation depth is typically used to describe the intensity variation of a laser or optical signal during the modulation process. Modulation depth, expressed in decibels (dB), is defined as the logarithmic representation of the ratio between the maximum and minimum values. The formula is as follows:

$$Modulation\ Depth\ (\text{dB}) = 10\log_{10}\left(\frac{T_{max}}{T_{min}}\right) \tag{S4}$$

According to the calculation results, the modulation depth reaches a maximum of 21.22 dB, when the wavelength is 1067.48 nm and the inner diameter is 0.04 μm. The following figure shows the modulation depth under different voltages when R_1 = 0.04 μm. Other structural parameters are consistent with the main text: the width of the nanobars w = 20 nm, the period of the unit structure P = 600 nm, the thickness of both the upper and lower silicon layers h_{Si} = 140 nm, the upper and lower Al_2O_3 layers $h_{Al_2O_3}$ = 5 nm, the thickness of the ITO layer h_{ITO} = 10 nm.

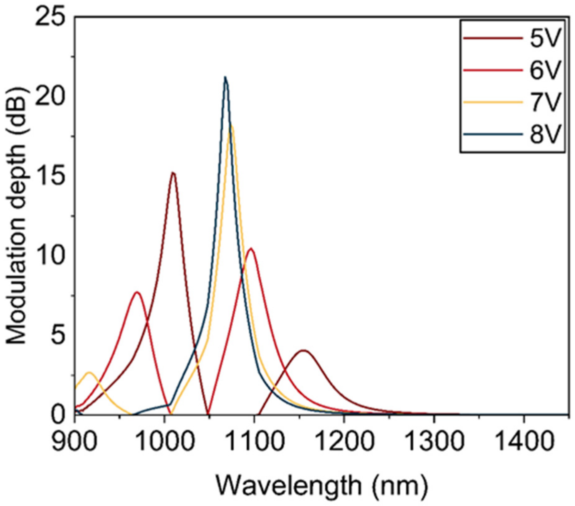

Figure S5. The modulation depth under different voltages when R_1 = 0.04 μm.

Table S1 shows the modulation depth of papers on spectral modulation of ITO applied voltage.

Table S1. Comparison of proposed and reported metasurfaces for dynamic regulation.

Structure	Materials	Wavelengths	Max modulation depth
**Cross-shaped array**[3]	Au/ITO	1200 nm-2400 nm	17 dB
**Slab**[4]	Au/graphene	800 nm-900 nm	16.81 dB
**Grating**[5]	Si/ITO	1300 nm-1700 nm	14.3 dB
**Disk**[6]	Si/ITO	1350 nm-1550 nm	41%
**Grating**[7]	Au/ITO	1300 nm -1750 nm	17 dB
**Grating**[8]	Si/ITO	1530 nm -1590 nm	86%
**Ring (this work)**	Si/ITO	900 nm - 1450 nm	21.22 dB

However, due to experimental errors, the modulation depth will be different from the theoretical value. In the experiment, the reasons for the possible errors are as follows: (1) The size of the ring structure is different from the theoretical value due to nano process technology. (2) As shown in Fig. S1, because the voltage is applied from one side of the metasurface, the resistance of the nanorings and nanobars may be too large to cause the nanorings structure to be subjected to different voltages at different locations (distance from the point where the voltage is applied).

Supplementary Note S6. The calculation of Si parameter and its effect on transmission spectrum.

First, we calculated variation of electron and hole concentration in the Si accumulation layer as a function of applied bias voltage and distance from the Si-Al_2O_3 interface.

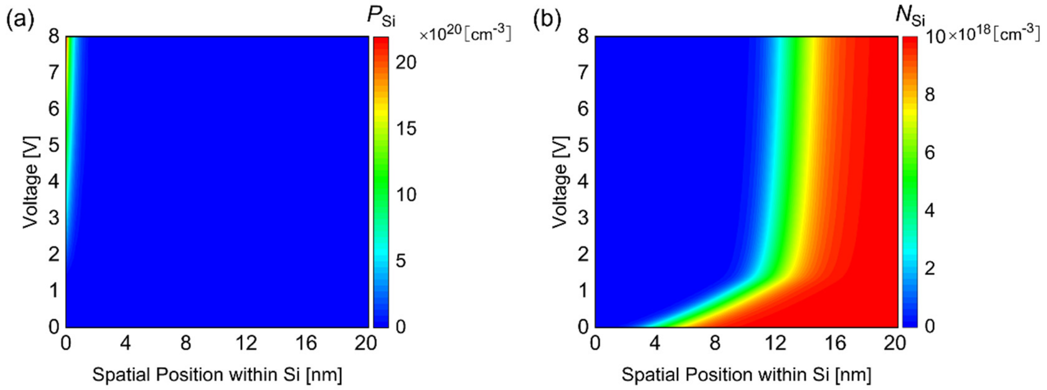

Figure S6. Variation of (a) hole concentration (b) electron concentration in the Si accumulation layer as a function of applied a bias voltage and distance from the Si-Al_2O_3 interface.

After solving Poisson equation and drift diffusion equation, the spatial distribution of carrier concentration within the active region of Si is transformed into a spatial distribution of dielectric constant using a dispersion model, which can be subsequently incorporated into optical simulations. The dielectric constant of Si can be obtained from the plasma–Drude model[9]:

$$\varepsilon_{\text{doped}}(\omega) = \varepsilon_{\text{undoped}}(\omega) - \frac{e^2}{\varepsilon_0 \omega}\left(\frac{N}{m_N^* \omega + \frac{ie}{\mu_N}} + \frac{P}{m_P^* \omega + \frac{ie}{\mu_P}} \right) \tag{S5}$$

However, comparing the cases with and without Si accumulation layer, the transmittance does not change significantly. There are two reasons for this analysis. First, when 8 V voltage is applied, the hole accumulation layer of Si is only 1 nm, which has little effect on the modulation of transmittance. At the wavelength range 900 nm to 1450 nm, the real part of the $\varepsilon_{\text{doped}}(\omega)$ does not approach 0. No electric field enhancement isachieved in the Si accumulation layer region.

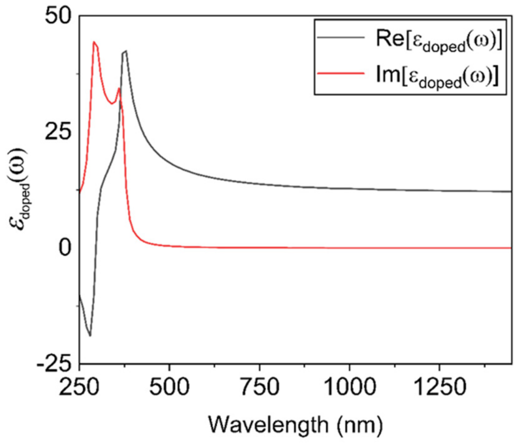

Figure S7. The real and imaginary parts of the Si permittivity located near the Si-Al_2O_3 surface at 8 V voltage.

Supplementary Note S7. Dispersion curve of ITO at the closest layer to the Al_2O_3 layer.

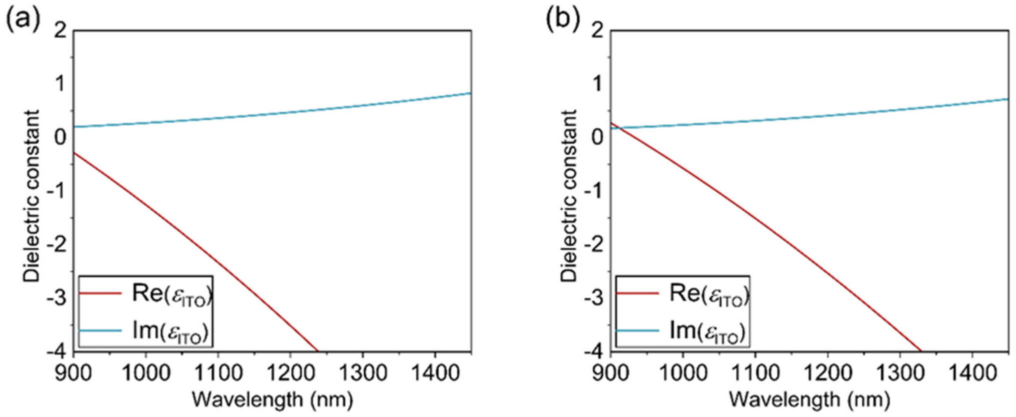

Figure S8. Dispersion curve of ITO at the closest layer to the Al_2O_3 layer (a) with a 0 V voltage and (b) with a 7 V applied bias voltage.

Supplementary References

1 Alaee, R., Rockstuhl, C. & Fernandez-Corbaton, I. An electromagnetic multipole expansion beyond the long-wavelength approximation. *Opt. Commun.* **407**, 17-21, doi:10.1016/j.optcom.2017.08.064 (2018).

2 Fan, S., Suh, W. & Joannopoulos, J. D. Temporal coupled-mode theory for the Fano resonance in optical resonators. *J Opt Soc Am A* **20**, 569-572, doi:10.1364/josaa.20.000569 (2003).

3 Xie, Z. T., Wu, J. Y., Fu, H. Y. & Li, Q. Tunable Electro- and All-Optical Switch Based on Epsilon-Near-Zero Metasurface. *IEEE Photon. J.* **12**, 1-10, doi:10.1109/Jphot.2020.3010284 (2020).

4 Shoaei, M., Moravvej-Farshi, M. K. & Yousefi, L. All-optical switching of nonlinear hyperbolic metamaterials in visible and near-infrared regions. *J Opt Soc Am B* **32**, doi:10.1364/josab.32.002358 (2015).

5 Kim, S. J. & Brongersma, M. L. Active flat optics using a guided mode resonance. *Opt. Lett.* **42**, 5-8, doi:10.1364/OL.42.000005 (2017).

6 Howes, A., Wang, W., Kravchenko, I. & Valentine, J. Dynamic transmission control based on all-dielectric Huygens metasurfaces. *Optica* **5**, doi:https://doi.org/10.1364/FIO.2018.FW6E.5 (2018).

7 Zhang, J. *et al.* Gate-tunable optical filter based on conducting oxide metasurface heterostructure. *Opt. Lett.* **44**, 3653-3656, doi:10.1364/OL.44.003653 (2019).

8 Forouzmand, A. & Mosallaei, H. Tunable dual-band amplitude modulation with a double epsilon-near-zero metasurface. *J Opt* **22**, 094001, doi:10.1088/2040-8986/aba03e (2020).

9 Palik, E. D. *Handbook of optical constants of solids*. Vol. 3 (Academic press, 1998).
